# Comment on “Analysis of the impact of pharmacological treatment on arterial stiffness in patients with connective tissue diseases”

**DOI:** 10.1016/j.ahjo.2026.100954

**Published:** 2026-10-13

**Authors:** Bandana Behera, Debasish Pradhan, Lalatendu Mohanty

**Affiliations:** a Pharmacy Science, Creighton University, Omaha, NE, USA; b UDPS, Utkal University, Bhubaneswar, Khurda, Odisha, 751004, India; c HNB Garhwal University, Uttarakhand, India

**Keywords:** Arterial stiffness, Connective tissue diseases, Pharmacological treatment, Pulse wave velocity, Rheumatoid arthritis, Glucocorticoids, IL-6 inhibitors, TNF-α blockers, Methotrexate, Cardiovascular risk

Dear Editor,

The review by Ramos-Becerra et al. [1], on drug treatment and arterial stiffness in connective tissue diseases makes firm statements in its abstract: IL-6 inhibitors and TNF-α blockers improve vascular outcomes, methotrexate does a little, glucocorticoids do long-term harm. We are not convinced the evidence, as presented, carries that much weight.

There is no account of how studies were found or chosen, and nothing on their quality. Even so, every drug class is given an evidence grade. Only statins are graded high, which by the authors' own definition needs several randomized trials or a consistent meta-analysis. But the meta-analysis they cite does not seem to have been confined to rheumatic patients, and what remains is a 6-week trial of 20 patients plus an 18-month study with no control arm. IL-6 blockade is a similar story. MEASURE, the largest placebo-controlled trial in the review, showed no gain in pulse wave velocity (PWV); the early fall was bigger on placebo. The positive studies either compared tocilizumab with another active drug or were small cohorts with no comparator. Not one study in the review counted cardiovascular events, so “improve vascular outcomes” overstates things [2].

What about glucocorticoids? In the rheumatoid arthritis papers cited under that heading they were given alongside conventional disease-modifying drugs, so their own contribution cannot be separated. For lupus the evidence is a single cross-sectional comparison with 22 treated patients listed, and patients on steroids for longer have usually been ill longer and more severely.

We also found places where the text and the summary of individual studies disagree. The 24-week randomized comparison of tocilizumab, etanercept and adalimumab lowered the cardio-ankle vascular index (CAVI) and augmentation index according to the text, yet the summary records a rise. For methotrexate alone in early rheumatoid arthritis the text gives a non-significant rise in PWV and the summary a fall. The lupus glucocorticoid study is summarized as finding lower carotid-femoral PWV in treated patients, with a remark that low doses may reduce stiffness, the opposite of what the review concludes. A pilot study of initial glucocorticoid therapy turns up in the summary as a 12-month comparison of biologics and JAK inhibitors. And in the IL-6 section five studies are cited under numbers that belong to unrelated references. We assume these are editing slips, but they involve the very drugs the conclusions turn on and should be corrected.

Then there is the grading itself. PWV, augmentation index, CAVI, intima-media thickness, and flow-mediated dilation are graded together, although the authors themselves explain that they differ [3]. Enalapril gets credit for reducing stiffness because CAVI fell in a trial where carotid-femoral PWV stayed the same. Almost all the treatment data are from rheumatoid arthritis, and for systemic sclerosis and the inflammatory myopathies, the review offers no drug study that measured stiffness. The title and abstract ought to say so. Nobody yet knows whether treating to PWV or CAVI changes what happens to patients, which makes the recommendation to bring them into routine practice premature [4].

## CRediT authorship contribution statement

**Bandana Behera:** Writing – review & editing, Writing – original draft, Visualization. **Debasish Pradhan:** Conceptualization, Data curation, Formal analysis. **Lalatendu Mohanty:** Writing – review & editing, Writing – original draft, Visualization.

## Informed consent

Not applicable.

## Ethical approval

Not applicable (Commentary on a previously published article).

## Declaration of Generative AI and AI-assisted technologies in the writing process

Generative AI tools were used only for language refinement and formatting assistance. All scientific interpretation, critical appraisal, and manuscript preparation were performed independently by the authors.

## Funding

None.

## Declaration of competing interest

The authors declare that there are no conflicts of interest.

## Data Availability

No new data were generated or analyzed in this study.
